# On Malignant Disease

**Published:** 1884-04-20

**Authors:** H. M. Lawson

**Affiliations:** Cuthbert, Ga.


### Southern Medical Record:

EDITORS:

THOMAS S. POWELL, M.D. R. C. WORD, M.D.

*R. C. WORD, M.D., Managing Editor.*

☞All Communications and Letters on Business connected with the RECORD must be addressed to the Managing Editor.

VOL. XIV. ATLANTA, GA., APRIL 20, 1884. No. 4.

### ORIGINAL AND SELECTED ARTICLES.

### ON MALIGNANT DISEASE.

By H. M. LAWSON, M.D., CUTHBERT, GA.

[The following article was first written for a prize in reply to the inquiry, "What is the proper line of study and experimentation likely to bring to light a successful treatment of malignant disease?" and was rejected, and—I think—rejected without trial. I now place it before the profession for it to decide as to whether or not the inquiry is answered; for I will not submit to the decision of any one man, or any half-dozen men, but I am content to abide the decision of the profession.]

I am as free from literary ambition as destitute of literary skill, and only take the pen in hand from a sense of duty; for I believe it to be the duty of all medical men to give the profession whatever beneficial discoveries they make. Believing this, I have strictly observed it during my professional life, and have written this under circumstances of great personal affliction, and hope that the plan of treatment proposed will be as successful in the hands of others as it has been in my own, and that it will not be condemned without trial. If, however, the profession rejects the method of treatment for fear of the remedy, I propose to go to any part of Georgia and treat a case under the supervision of a committee appointed by any of its medical faculties, or the State Medical Association, and convince them that the treatment is both efficient and harmless.

When I began the study of materia medica, I found that there was quite a diversity of opinion among medical authorities concerning arsenic, some saying that its external use was murderous, others safe, others, again, proposing to reject it as a remedy altogether. On entering the profession and discussing the remedy with medical men, I found that but few used it, and these rarely, otherwise than as a dernier resort, and that all were, more or less, afraid of it; so I determined to examine the remedy for myself, but deferred to do so, partly for want of an opportunity; again, excusing myself from the task by saying: "Surely, if the majority of the profession wish to ostracize it, there must be ample ground for their so-doing." Finally, on reading an article in a medical journal, "The Arsenic-eaters of Styria," I determined no longer to defer investigating the subject, and the result of that investigation will be found in the following pages.

I was aware that many cases of malignant disease did well on arsenic while it could be taken, but in consequence of the supervention of its cumulative effects, it had to be suspended before all the benefit it seemed capable of affording could be brought to bear upon the case. The article referred to convinced me that there was a way to give arsenic continuously without hindrance from the ill effects alluded to; and I began experimenting to ascertain the fact. My first subject of experiment was myself. I had not continued my experiments long before I discovered the fact that if the dose of arsenic was large enough to prove laxative the cumulative effect would not occur, and I became very anxious for a suitable case of malignant disease upon which to test the treatment. Before this, while studying the subject of malignant disease, I became convinced that, if ever a successful plan of treatment for that intractable malady was discovered, arsenic would play an important part therein. It was some years after this before my first case, the first one, reported at the conclusion of this article, presented itself.

TREATMENT.—Preparatory to commencing an arsenical course, the condition of the patient's bowels should be ascertained. If they are moved daily, or in a healthy condition, the patient may be at once put upon the remedy; but if they are more or less constipated, care should be taken to correct this condition. This is best done (according to my experience) by giving a laxative dose of rhubarb every night for two or three nights before commencing with the arsenic, omitting, however, to give it the night before commencing the arsenical course, so as to be certain that the first dose of arsenic produces the due effect on the bowels. I would say, in reference to rhubarb, in this connection, that it is far superior to any other remedy as a laxative used with arsenic, and will be found of great service at the commencement and termination of the arsenical course.

*The way to give arsenic, in malignant disease, is in laxative doses, and once only in twenty-four hours; and the time is in the morning fasting.* It should be given in half a cup of coffee, as warm as it can be taken. If the bowels are not moved within three hours a laxative dose of rhubarb should be given and repeated every three hours until the bowels move.

*One-eighth of a grain of arsenious acid will generally prove laxative*; therefore, it is a good dose to begin with; but, should it fail to impress the bowels, give the rhubarb as already directed, and on the next morning *give one-fourth of a grain*, and should this dose fail to prove laxative (which it will rarely do), pursue the same treatment with the rhubarb as before, and so on, until the laxative dose of arsenic is arrived at. The laxative dose found, it is to be given every morning throughout the treatment.

After taking the remedy for a short time, the dose found to be laxative will require to be augmented: the bowels becoming accustomed to that quantity, a sufficient laxative effect will not otherwise be maintained.* When this occurs, add *one-eighth of a grain to the next dose given, and the laxative effect is to be maintained by adding the last-mentioned quantity as often as the case requires during the treatment.* This augmentation may be safely continued until the dose reaches two grains, and few cases, if any, will ever require a larger dose than one grain.

In from three days to three weeks, all pain and other morbid sensations will cease in the diseased part, which indicates the arrest of the disease, and it is manifestly proper to defer surgical interference until its growth is arrested, although it should require a longer time than that mentioned. When the pain or other sensations cease, the diseased part should be removed by surgical operation; but in cases where surgical operation has already been performed, or in other cases where sparseness of tissue forbids the use of the knife, a solution of the chloride of zinc, ten grains to the ounce of water, increased if necessary, so as to produce a profuse discharge, will be found a serviceable resource. A caustic treatment, such as that just mentioned, is, in my limited experience, preferable to the stronger applications often used in such

---

* Note 1.

cases. My experience is confined to the use of caustics only in connection with the internal use of arsenic.

As soon as the morbid structures have melted down under the action of the caustic solution, it should be discontinued, and a healing application should be substituted. I have found new clean tallow superior to anything else as a dressing in these cases.

*The arsenic should be continued in all cases until the parts are healed, and for a week afterward.*

In withdrawing the remedy, especially if it has been found necessary to continue it longer than six weeks, or when it was necessary to increase it several times, it is not well to discontinue it too suddenly. My plan is to reduce to the original laxative dose, and give that every other morning for three or four days; but continue the coffee every morning (which will act almost as promptly as if it contained the arsenic), then discontinue the remedy altogether; but continue the coffee every morning for a week. Care must be taken to keep the bowels gently open during the entire time of withdrawing the remedy, and it is in doing this that rhubarb is found so valuable.

To resume. To put the patient upon laxative doses of arsenious acid, and to maintain the remedy at its laxative dose, are the two important points that demand attention, since they secure the full tonic and alterative effects of the remedy, and, at the same time, its elimination.*

A short account of the effects of arsenic used in this way will not be out of place here. The first dose will be apt to produce nausea, and sometimes vomiting, attended by slight pains in the stomach, and griping pains in the bowels, somewhat like the effects of a drastic cathartic, but not so severe; but these effects are confined to the initial dose, the succeeding ones producing no unpleasant effects, but rather the contrary, nor is any further disturbance experienced, unless it becomes necessary to increase the dose; but after every such increase the same phenomena are produced, but in a slighter degree. All these unpleasant effects cease as soon as the bowels are moved, and are succeeded by a keen appetite and a feeling of excitement.

I am well aware that this method of giving arsenic is at variance with the generally received mode, as is, also, the time of giving it; but if the way recommended be the best, I feel quite sure that it will be recognized and adopted when experience has so determined, to which arbiter I am content to refer the disputed point.

* Note 2.

I do not claim to present the profession with a new remedy for malignant disease; for every one acquainted with the subject is aware that arsenic has been used from very early times for cancerous affections; but I am only offering a new, safe and, as I believe, successful method of using an old one: a way, too, in which it can be continuously given as long as the circumstances of the case require.

Experience has fully satisfied me that arsenic is not half so unmanageable a remedy as it is believed to be by the majority of the profession, if its peculiarities are understood. It is true the circumstances under which it becomes dangerous, the medicinal agents and articles of diet with which it will not agree, must be known and avoided. The following I have found more or less detrimental. All astringent remedies, especially opium and its preparations,* alcoholic liquors, mercury and its preparations, tobacco and all constipating articles of diet; for the fact should never be lost sight of that it is arsenic *retained* in the system that does harm, but when *eliminated* no harmful effects will ensue, unless the dose given considerably exceeds those herein recommended or the articles above mentioned as detrimental either constipate the bowels or otherwise interfere with the due effects of the remedy.

The arsenic must all pass out of the system, or its cumulative effects will ensue; and that it passes off, and chiefly by the bowels,† I have reason to be assured. I have seen some reference to the ill effects of arsenic on the stomach, or permanent injury to the nervous system; but I have, after a long experience with the remedy, in malignant as well as non-malignant diseases, witnessed no such effects; and, judging from my own experience, I am forced to believe that, when such effects are induced, it is in consequence of the ignorance of the rules I have given for their avoidance.

Of course, I do not accuse any one of willful or criminal ignorance, but only that the obscurity of the subject rendered it necessary for some one to institute the experiments that I have attempted before any one could possibly understand the peculiarities of the remedy sufficiently to avoid its injurious effects.

There is certainly a line which separates between the curable and the incurable cases, but in my experience the remedy has always indicated this. If the pain is relieved or ameliorated from the preparatory course, i. e., the first three weeks of treatment, it is a curable case, but should it fail to have this effect the result will be doubtful.

---

* Note 3. † Note 2.

I believe in the curability of almost all recent cases where the aid of the knife or other methods of removal are not impracticable, but experience has yet to prove this, and also to determine to what extent cases in which the disease is beyond the reach of surgical interference are relievable.

I have taken ample time to test this method of using arsenic, but am not sure that I have used the preparation best adapted to the treatment. I have used arsenious acid* exclusively, and it has answered the purpose fully; at all events, the cases treated by it are still free from malignant disease and in good health otherwise, although several years have elapsed since their treatment.

CASES.—The first case that afforded me an opportunity of testing arsenic in malignant disease was Mrs. T., aged fifty-six years. She was of spare habit and decidedly delicate, quite the reverse of what is understood by the term robust.

A large, ragged, wart-like growth, with an intensely red base, had made its appearance on her nose near its middle, attended by a burning sensation and lancinating pain; and, as everything connected with the case was decidedly malignant, and her bowels being in a proper condition, I began the treatment by putting her upon one-eighth of a grain of arsenious acid, at the time and in the manner already detailed. The dose proving laxative, it was continued for three days; but, as no apparent benefit resulted, she expressed a wish to have the growth removed. To this I objected, telling her that if it was malignant she ought to continue the constitutional treatment at least two weeks, and that if she did so, in all probability it would not return after removal, but an earlier removal would almost certainly be followed by a return of the disease, while if it was not malignant no operation was necessary. This seemed to satisfy her, but, being afterward counseled by her friends, her fears were re-established, and she left next day for a neighboring city, without further consulting me, and had the operation performed. After her return I heard nothing from her for a week, when I received a note from her, requesting me to call and see her. On my arrival she met me at the door, saying: "Doctor, I am sorry I did not follow your advice. I have had the operation performed, and the disease has returned, as you predicted, and I wish you to resume the management of the case." Had it been any other kind of a case I should have had nothing more to do with it; but, as it offered me a long-wished-for opportunity of testing the arsenical treatment, I consented, at the same time tell-

* Note 5.

ing her that I could not now promise her relief, but if she would be guided by me alone in regard to the case for the future, I would do my best for her. To this she gladly consented, and I resumed the treatment by putting her upon the arsenic in the same dose as at first. One increase of the remedy was required in a six week's treatment, at the end of which time the parts were healed, and the only evidence of the malady remaining was a pit about the size of a split pea. In this case no external application was made,* nor was any other remedy but the arsenic used; neither was any further surgical operation required. Several years have elapsed without any indication of the disease returning. This lady visited me a few days ago and is still free from all evidence of malignant disease, and in good health.

Shortly after the above case was discharged, a lady seventy years of age applied for treatment. The greater part of this patient's nose and cheeks were destroyed by a lupus of twenty years standing. On her temples and what remained of her cheeks were several black, rough spots, from the size of a five cent piece to that of a dime. The age of this patient, as well as that of the disease, precluded the possibility of a cure, and I at once told her so, but as she was anxious for me to make an attempt for her relief, I agreed to put her upon a three weeks treatment merely to gratify her, without any hope myself of doing her the slightest good. At the expiration of that time, although there was no apparent benefit to the original disease, the black spots above mentioned had all disappeared, leaving the skin in a healthy condition. These spots, she assured me, were exactly like the one that twenty years before began her disease, and a cure, I am satisfied, would have been the result had the treatment been instituted at an earlier period. Her advanced age prevented my pressing the treatment, as I should have done with a younger and more vigorous patient, so the treatment was discontinued.

The case now to be reported is one of great interest, showing in a remarkable manner the benefit as well as the harmlessness of arsenic as herein advised, and the singular behavior of the tumor while under treatment.

This patient had a firm, but small tumor, with an irregular surface, located midway between the eye and the upper lip, attended by lancinating pain and a peculiar sensation as of the gnawing and crawling of ants—as the patient expressed it. This one was of fifteen years standing, and had, during that time, been under

---

* Note 4.

several physicians, and two or three "cancer doctors," so-called, and the tumor had been removed two or three times by caustic agents, but had promptly returned. There was no diversity of opinion as to the nature of this tumor among the medical gentlemen who had seen or attended the case, all agreeing as to its malignancy.

I put this patient upon one-eighth of a grain of arsenious acid, in the manner before described. Very soon after the beginning of the treatment the pain and the other unpleasant sensations mentioned ceased. After the third month I found it necessary to increase the dose to one-fourth of a grain, but no farther increase was found necessary, and, had the patient submitted to the necessary operation for the removal of the disease at the proper time, the cure would have been speedily accomplished; but, the patient objecting to this, the treatment was continued, hoping that ulceration and absorption would effect its removal, for during the fourth month the tumor became deeply grooved in the following manner: A deep groove opened the tumor vertically, and another horizontally, crossing each other in the center, and from which a profuse discharge occurred, diminishing the size of the tumor considerably. At the end of the seventh month it healed, leaving a flat, painless tumor, about one-fourth its original size. At the close of tenth month I discontinued the treatment, thinking that a cure could not be effected without an operation; but I was agreeably surprised to find that the tumor continued gradually to diminish until the parts returned to the natural condition, and, although several years have elapsed, there is not the slightest indication of a return of the disease, and the patient is in perfect health in every respect.

The only ill effects I have ever seen from arsenic occurred in this case, and were occasioned as follows: After the patient had been under treatment about four months, business compelled me to leave home for a few days. (Up to that time I had seen the patient daily.) Before leaving, I prepared and left with the patient three doses, as I only intended to be absent three days. During my absence the weather became unsettled, and the patient, fearing I would be detained longer than the time specified, made four doses out of the last two, and when I returned, which I did in due time, I found the patient cumulatively poisoned, at which I was greatly surprised, but was relieved on being told about the dividing of the doses, and the bowels not having been relieved for a day or two, this accounted for the unexpected effects. Believing that to open the bowels with rhubarb, and to resume the arsenic in one-fourth of a grain doses, was the best way out of the difficulty, I did so, and in forty eight hours the cumulative effects had disappeared.

Here was a patient who had taken arsenic ten months, and is still free from any ill-effects from it; but I shall leave all comment to others.

I know very little about rabies or yellow fever practically, but wish to offer the following suggestion as to their prophylaxis and treatment, for if I were bitten by a rabid animal, or had I a patient so bitten to treat, I should cauterize the wound, but I should, all the same, put myself or my patient upon arsenic in laxative doses, and continue the remedy at least three months, or if I had to face an epidemic of yellow fever I should take the same remedy in the same manner as long as I was exposed to the disease; and nothing but its failure would convince me that it would not prove prophylactic in both cases.

It does not require any great deal of professional prescience to infer that a plan of treatment that will cure even mild cases of malignant disease would also successfully meet that class characterized by the term, "semi-malignant;" also, in all chronic diseases of a non-malignant nature to which the remedy is applicable. I have found it much more efficient when given as above advised; in fact, it will be found capable of meeting successfully many cases that bid it defiance in the doses usually administered.

If a menstruating woman takes arsenic, the menstrual flow will be arrested. Experience must determine whether or not such cases are to be denied the benefit of the arsenical treatment. I know nothing of such cases from experience.

NOTES.

NOTE 1 —When the dose loses its laxative influence, some patients complain of depression, and often of cephalalgia. If severe, a dose of rhubarb may be given, and as soon as the bowels move the unpleasant symptoms generally cease, and the augmentation which must be made on the next morning will prevent any recurrence of these unpleasant symptoms until further increase of the dose is necessary.

NOTE 2.—Arsenic taken as directed is chiefly eliminated by the bowels. This was not found out by chemical analysis, but by a way almost as positive, if not as exact. There is a coleopterous insect common to Georgia which infests privies, barnyards and other places where fecal matter is deposited, and feed upon it. Now, when patients under arsenic relieve their bowels at such places these insects die by the hundred. I have noticed this so often that I cannot be mistaken as to the cause. That arsenic is also eliminated by other secretory organs, I know from the fact that if a nursing woman takes oneeighth of a grain the bowels of the infant are as promptly affected as the nurse's, and I have for some years taken advantage of this fact by treatiug children for intermittent and other diseases, to which tbe remedy is applicable, by giving it to the mothers.

NOTE 3.—It is to be regretted that opium and its preparations are so obnoxious to the arsenical treatment, since almost all cases that are at all advanced find it necessary to alleviate their sufferings by them; but before even an attempt at a cure by the arsenical treatment is possible, all the preparations of opium must be wholly discontinued. Conium, hyoscyamus and belladonna can all be used with arsenic; but a dose of opium given to a patient under the arsenical treatment will act exactly as a poisonous dose of arsenic. M. Dewy gives the preference to conium. He says (in Med. Gaz. de Lyons, No. 52): "A properly made extract of conium will afford more prompt relief, and is preferred by cancer patients to any other remedy." I have nothing to offer from experience, and should try the conium if I had a patient requiring a narcotic, and I should discontinue even this as soon as the treatment rendered it possible.

NOTE 4.—Except the tallow. This patient, as well as the others which I have treated, was put on the best hygienic regulations possible. They were also directed to take the hot and cold bath just after taking the arsenic; and to take exercise in the open air: this last, the arsenic strongly disposes the patient to do.

I believe summer to be the best time to treat cancer, although some of my patients did well in the winter. I allowed my patients full diet, and to consult their appetite as to what was eaten, *when not manifestly improper*.

NOTE 5.—For convenience, I now use a syrup of arsenic, made by adding to boiling simple syrup one grain to the ounce.

